# Nasal lymphatic obstruction of CSF drainage as a possible cause of Alzheimer’s disease and dementia

**DOI:** 10.3389/fnagi.2024.1482255

**Published:** 2024-10-21

**Authors:** William Thomas Phillips, Joyce Gensberg Schwartz

**Affiliations:** ^1^Department of Radiology, UT Health San Antonio, San Antonio, TX, United States; ^2^Department of Pathology, Methodist Hospital, San Antonio, TX, United States

**Keywords:** dementia, parasympathetic activity, glymphatics, tau, amyloid, Alzheimer’s disease, metabolic syndrome, CSF drainage

## Abstract

Alzheimer’s disease, the most common form of dementia among older adults, slowly destroys memory and thinking skills. In recent years, scientists have made tremendous progress in understanding Alzheimer’s disease, still, they do not yet fully understand what causes the disease. This article proposes a novel etiology for Alzheimer’s disease. Our hypothesis developed from a review of nuclear medicine scans, in which the authors observed a significant increase in nasal turbinate vasodilation and blood pooling in patients with hypertension, sleep apnea, diabetes and/or obesity, all risk factors for Alzheimer’s disease. The authors propose that nasal turbinate vasodilation and resultant blood pooling lead to the obstruction of normal nasal lymphatic clearance of cerebrospinal fluid and its waste products from the brain. The nasal turbinate vasodilation, due to increased *parasympathetic* activity, occurs alongside the well-established increased *sympathetic* activity of the cardiovascular system as seen in patients with hypertension. The increased parasympathetic activity is likely due to an autonomic imbalance secondary to the increase in worldwide consumption of highly processed food associated with dysregulation of the glucose regulatory system. The authors’ hypothesis offers a novel mechanism and a new paradigm for the etiology of Alzheimer’s disease and helps explain the rapid worldwide rise in the disease and other dementias which are expected to double in the next 20 years. This new paradigm provides compelling evidence for the modulation of the parasympathetic nervous system as a novel treatment strategy for Alzheimer’s disease and other degenerative brain diseases, specifically targeting nasal turbinate lymphatic flow.

## 1 Introduction

In 1906, Alois Alzheimer described “a peculiar disease” in the case of Auguste D., a patient with profound memory loss, unfounded suspicions about her family, and other worsening psychological changes (Alzheimer, 1907). In her brain at autopsy, he reported dramatic shrinkage and abnormal deposits in and around nerve cells (Davos-Alzheimer’s-Collaborative, 2024). The term ‘Alzheimer’s disease’ has been used for over 100 years since first used in 1910. However, it was not until the 1990s that neuropathologists settled on a definition of Alzheimer’s disease (AD) based entirely on a sufficient burden of extracellular amyloid neuritic plaques and intraneuronal neurofibrillary tangles at postmortem examination (Knopman et al., 2019). The soluble building blocks of these structures are amyloid-beta peptides for plaques and tau for tangles.

Although there now exists agreement regarding the histopathologic findings needed to diagnose a patient with AD, there has been no agreement regarding the origination and development of the disease.

Researchers are becoming aware that amyloid and tau deposition in AD frequently coexists with vascular disease. It is now realized that mixed pathology dementias account for more than 50% of total dementia cases with amyloidosis and vascular disease being the most frequent combination of AD (Carey and Fossati, 2022). In recognition of this common occurrence of mixed disease, the American Heart Association and the American Stroke Association have introduced the concept of Vascular Cognitive Impairment (VCI) to capture the entire spectrum of cognitive disorders associated with all forms of cerebral vascular brain injury (Gorelick et al., 2011).

Numerous hypotheses regarding the pathogenesis of AD have been proposed including the effects of amyloid plaques to alter neurological function. Previous clinical trials, however, focusing on removing amyloid deposits from the brain, have been disappointing (Anderson et al., 2017). Recently, however, several drugs that targeted amyloid removal have shown a mild slowing in the rate of AD’s progression and have been approved by the Federal Drug Administration (FDA) (Li et al., 2024).

### 1.1 The hypothesis

This paper focuses on areas not previously considered in the pathogenesis of AD. The authors hypothesize an increase in parasympathetic activity in the nasal turbinates leads to obstruction of the cerebrospinal fluid’s (CSF) normal nasal lymphatic drainage containing the waste proteins tau and amyloid. The obstruction or blockage of the CSF drainage results in the accumulation of these waste proteins in the brain. The increased *parasympathetic* activity of the nasal turbinates occurs simultaneously with the well-established increase in *sympathetic* nervous activity of the cardiovascular system in conditions that are known risk factors for AD including hypertension, diabetes, and obesity.

## 2 Evidence for the hypothesis: critical evaluation and discussion

The incidence of AD increased by 140% between 2000 and 2020 (Institution, 2024). The disease is more common in women, who have a greater than 50% chance of developing AD versus men (Pszczołowska et al., 2024). In addition, AD is not equally distributed in populations worldwide. In the United States, there are racial disparities in the incidence of AD with Hispanic individuals having 1.5 times and non-Hispanic Black individuals having 1.27 times greater incidence of AD than non-Hispanic White individuals (Churchill et al., 2024). The cost of treatment and care of AD patients is very high with unpaid dementia caregiving valued at $346.6 billion in 2023. The total payment in 2024 for healthcare, long-term care, and hospice services for people aged 65 years and older with dementia in the United States is estimated to be $360 billion (Institution, 2024).

### 2.1 The obesity epidemic

In high-income countries today, individuals consume greater than 50% of ultra-processed food in their diet (Dai et al., 2024). These foodstuffs include packaged chips, soda and energy drinks, and ready-to-heat-and-eat meals. They are thought to be an important driver of the obesity epidemic, in part because they seem to make us eat more (Ouyang, 2023).

The obesity epidemic occurring in the United States has also been noted in other developed and developing countries throughout the world. Changes in dietary patterns in China, with increased consumption of refined grains and highly processed, high-sugar, and high-fat foods, continue to grow. At the same time, physical activity levels in all major regions of China have decreased (Pan et al., 2021). In China, the number of processed foods available was four times higher in 2013 than in 1999 for a 22.4% annual growth over the 15 years. Over half of the packaged foods sold in China’s markets are processed foods. Overweight, obesity, hypertension, and metabolic syndrome in the Chinese population have become serious public health problems. A recent report stated China had the highest number of overweight and obese children globally (Pan et al., 2021). The increased rate of obesity and hypertension in China likely explains the fact that stroke is now the number one cause of death in China (Tu et al., 2023).

### 2.2 Clinical observations during nuclear whole-body blood pool scans

One of the authors of this article, a nuclear medicine physician, observed unique findings during the performance and review of several hundred whole-body bone scans performed on patients who had been referred to the Nuclear Medicine Department. The observations occurred during the first phase of a two-phase bone scan. The first phase of the scan, known as the “blood pool,” is performed to find sites of increased vascularity due to inflammation. It is a scan of the whole body and is obtained within the first 7 min after injection of the bone imaging radionuclide while it remains in the blood. The second phase of the scan is performed 3 h following injection of the radionuclide, when the bone imaging agent has deposited into the bone.

While performing and interpreting the first phase of the whole-body blood pool scan, significant uptake was observed in the nasal turbinate region of those patients with pre-existing conditions of hypertension, diabetes, and/or obesity, all risk factors for AD. It was these scans that led the authors to develop a new hypothesis for the etiology of AD.

## 3 No single etiologic mechanism has been identified for AD

Many investigators ascribe the mechanisms of AD and dementia to multiple factors including interactions between diet and lifestyle (Khemka et al., 2023). Amyloid and tau accumulation as an etiology of AD has been proposed as well as other etiologies including cerebral insulin resistance and glucose hypometabolism (De La Monte, 2016; Mullins et al., 2017; Neth and Craft, 2017), and synaptic dysfunction and the role of mitochondrial dysfunction with alterations in intracerebral adenosine triphosphate (ATP) levels (De La Monte, 2016; Cenini and Voos, 2019). There is also evidence that AD is linked to obesity, diabetes, and the Western diet (Arvanitakis et al., 2004; Gomez-Pinilla and Yang, 2018). The contribution of genetic factors is known to be associated with AD with APOE4 homozygosity significantly increasing the risk for the disease. APOE4 homozygotes are estimated to have a 60% chance of developing AD dementia by age 85. Although APOE4 homozygotes account for only ~2% of the overall population, they make up ~15% of AD cases (Fortea et al., 2024; Xu et al., 2024).

Different theories about the predisposition to the development of AD have been proposed, including perinatal influence (undernutrition) (Gauvrit et al., 2022), socioeconomic status factors (Wang et al., 2023), low education (Li et al., 2023), hypertension (Carey and Fossati, 2022), hyperhomocysteinemia (Van Dam and Van Gool, 2009), obesity and insulin resistance (Terzo et al., 2021), diabetes (Janoutová et al., 2022), depression (Diniz et al., 2018), ultra-processed food consumption (Claudino et al., 2023), and smoking (Durazzo et al., 2014). Although there are many well-known risk factors for AD, there is no clearly identifiable cause.

A lack of understanding of the mechanism of AD contributes to the fact that an estimated six million individuals live with dementia today in the United States with no hope of a cure but with medications and management strategies that can only temporarily slow the progression of the disease. A possible explanation for this overall lack of understanding regarding the etiology of AD is that the underlying basic pathophysiology leading to its development is not being addressed. There is a need to develop new paradigms for understanding Alzheimer’s disease development which could lead to the development of new approaches to therapy.

## 4 Risk factors for AD and vascular dementia related to metabolic syndrome

Increasing evidence has emerged to suggest that AD is multi-factorial with vascular pathology working together with amyloid-beta and tau to produce cerebral pathology and cognitive decline (Carey and Fossati, 2022).

Well-recognized risk factors for this multifactorial process are associated with metabolic syndrome and are *considered to be modifiable* with the potential to reduce dementia. These risk factors are reviewed as follows:

### 4.1 Hypertension

Multiple longitudinal studies have found that midlife hypertension is associated with an increased risk of AD and dementia (Launer et al., 2000; Carey and Fossati, 2022).

### 4.2 Obesity

Obesity and increased visceral fat have been associated with reduced cortical thickness and brain shrinkage (Veit et al., 2014). Increased fat deposits in the abdominal region have also been related to lower cognitive function in middle-aged males (Golan Shekhtman et al., 2024). A higher body mass index (BMI) and insulin resistance have also been associated with lower cortical thickness and brain shrinkage in the bilateral temporal poles (Dolatshahi et al., 2023).

### 4.3 Diabetes and glucose intolerance

Diabetes and glucose intolerance, both components of metabolic syndrome, are associated with a significantly increased risk for all types of dementia. A meta-analysis showed a 73% increased risk of developing all types of dementia with a 56% increase in AD and a 127% increase in vascular dementia in patients with diabetes (Gudala et al., 2013).

### 4.4 Consumption of highly processed foods

Increased consumption of highly processed food has been linked to the development of AD (Claudino et al., 2023). In a prospective cohort study of 72,083 participants, ultra-processed food was a significant contributor to the development of dementia. Importantly, a 10% reduction in the consumption of ultra-processed food was estimated to be associated with a 19% reduction in the development of dementia (Li H. et al., 2022).

### 4.5 Lack of exercise

Low cardiorespiratory fitness in obese patients has been associated with decreased cognitive function as compared with obese patients with high cardiorespiratory fitness (Wichayanrat et al., 2022).

### 4.6 Sleep disturbances and obstructive sleep apnea

Sleep disorders are common in AD and have previously been considered to be caused by a progression of the disease. It has been realized in recent years, however, that there is likely a bi-directional relationship between sleep disorders and AD. Considering the importance of sleep to brain health, sleep disorders may well be contributing to AD’s pathology as well as AD contributing to the sleep disorder (Borges et al., 2019).

Obstructive sleep apnea has also been causally linked to the development of AD and other neurocognitive disorders as well as cardiovascular disease (Cavaillès et al., 2024). In a meta-analysis of 11 studies comprising 1,333,424 patients, those with sleep apnea were found to have a significantly increased risk of neurocognitive disorders (Guay-Gagnon et al., 2022). The hazard ratios (HR) associated with sleep apnea are as follows:

HR: 1.43 [95% CI 1.26–1.62] for any type of neurocognitive disorder.

HR: 1.28 [95% CI 1.16–1.41] for AD.

HR: 1.54 [95% CI 1.30–1.84] for Parkinson’s disease.

## 5 Measures to decrease the risk of developing dementia

The altering of various lifestyle measures has been recognized as a way of decreasing the risk of developing dementia.

### 5.1 Exercise

Many prior studies have shown that a healthy lifestyle decreases the risk of developing all forms of dementia. The preventative lifestyle measures include a healthy diet and an increase in exercise (Wang et al., 2023). Exercise in all forms, particularly vigorous exercise, is associated with a decreased risk of AD (De la Rosa et al., 2020; Yu et al., 2020). Aerobic exercise (with an intensity of 50–75% of VO2 max) prevents hippocampal volume reduction, spatial memory reduction, and learning reduction through increasing synaptic flexibility (Pahlavani, 2023).

### 5.2 Healthy diet

Consuming an increased amount of fruits and vegetables has been shown to slow cognitive decline (Haskell-Ramsay and Docherty, 2023). Cognitive protection has been particularly positive for diets that include green leafy vegetables. One study has shown that patients eating a diet that included at least one serving of green leafy vegetables per day had significantly slower cognitive decline which was the equivalent of being 11 years younger in age (Morris et al., 2018).

Several specific diets have been recommended to decrease the risk of dementia development including the following:

#### 5.2.1 Mediterranean diet

Dietary interventions, such as the increased consumption of vegetables, have been associated with a decreased risk of AD development (Guzzi et al., 2016). Several studies have shown that a Mediterranean diet will decrease the risk of developing AD (Hoscheidt et al., 2021; Stefaniak et al., 2022).

#### 5.2.2 MIND diet

The Mediterranean-DASH Intervention known as the MIND diet is a hybrid of the Mediterranean diet and the DASH (Dietary Approaches to Stop Hypertension) diet with modifications to include foods that have been putatively associated with a decreased risk of dementia which was first reported in 2015 (Morris et al., 2015). In a study of 960 participants in the Memory and Aging Project, the MIND diet was found to be positively associated with a slower decline in global cognitive function (*p* < 0.0001) (Morris et al., 2015). A systematic review of 13 articles in 2022 investigating the MIND diet found that the MIND diet was superior to other plant-rich diets for improving cognition (Kheirouri and Alizadeh, 2021). However, a more recent 2023 study found that cognitively unimpaired participants with a family history of dementia did not differ significantly between those who followed the MIND diet and those who followed the control diet with mild caloric restriction (Barnes et al., 2023). This may indicate that the most significant aspect of all of the protective diets may be mild caloric restriction.

## 6 Methods

### 6.1 Nuclear scans

**Figure 1:**
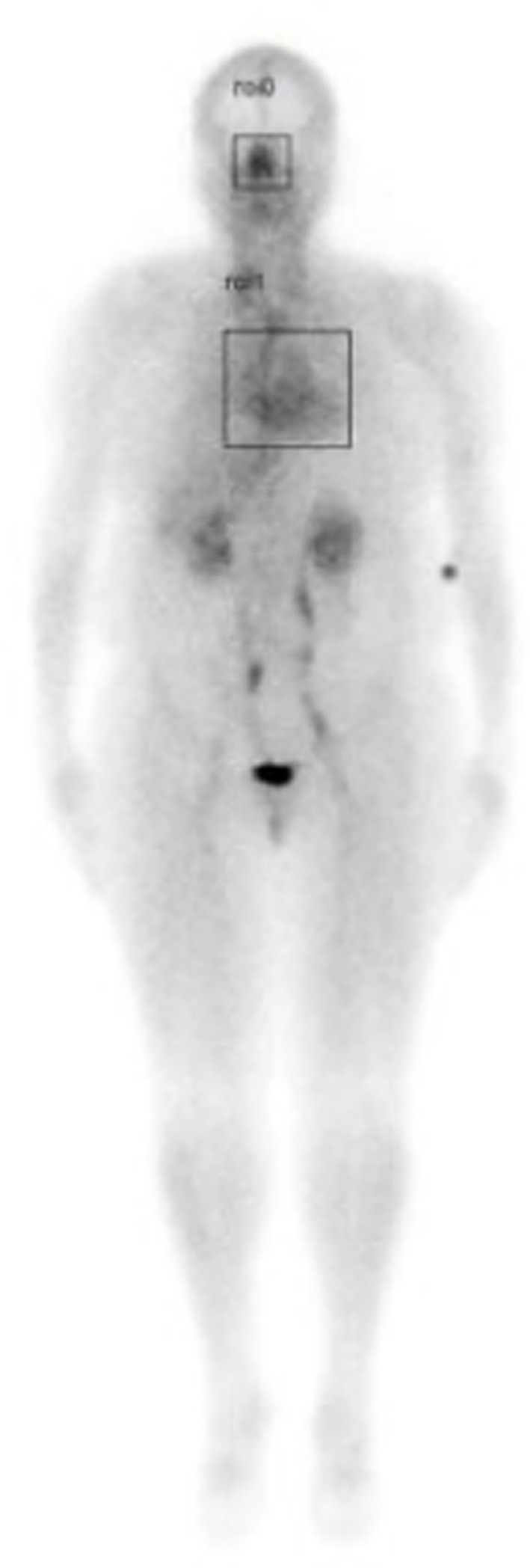
Illustration of whole-body scan showing boxed areas (nasal turbinates and cardiac) analyzed for maximum pixel counts to determine nose/heart max ratios.

A retrospective study was performed by the authors which included a review of whole-body nuclear scans from 200 patients who had been referred to the Nuclear Medicine Department at the University of Texas Health Science Center at San Antonio over 3 years, from May 1, 2017, until May 1, 2020. In this retrospective study, quantitative analysis of the *nasal turbinate* blood pool was compared with the *cardiac* blood pool using region of interest analysis by measuring the maximum pixel counts in each region as shown in Figure 1.

With scintigraphic imaging, it is possible to determine the distribution and activity of blood in the nasal region as compared to the cardiac region. Nose/heart ratios were determined by placing a square region of interest box over the area of the nose on the nuclear scan. The activity in maximum pixels was determined in each box, and a ratio of the maximum pixels in the nose was divided by the maximum pixels in the heart. Using the maximum pixel activity is very similar in technique to analyzing the maximum standard uptake value (MaxSUV) as determined in PET imaging for monitoring cancer metabolism. The use of a box and maximum pixel activity decreases the subjectivity of the image analysis incurred as compared to drawing an outline around the whole organ.

Whole-body scintigraphic images were acquired during the 7-min interval immediately following injection of a bone avid radiopharmaceutical, technetium-99 m methylene diphosphonate (^99m^Tc-MDP) before it had time to begin accumulating in the bone (Phillips et al., 2022). Images were obtained with a dual-headed gamma camera (GE Infinia Hawkeye 4, Boston, MA) using low-energy, high-resolution collimators with an energy window set at 140 keV and with a 20% energy window moving at a rate of 36 cm/min (Phillips et al., 2022).

### 6.2 Review of charts

Two hundred patient charts were reviewed from patients referred to the Nuclear Medicine Department for a two-phase whole-body bone scan. The first phase includes a whole-body blood pool scan (Phillips et al., 2022). Information obtained from patients’ charts included BMI, glucose level/diabetic status, blood pressure, history of sleep disorders, cardiovascular disease, hemoglobin A1c (HbA1c), and prescribed medications.

### 6.3 Population studied

Greater than 50% of the patients studied were Hispanic individuals, similar to the population of San Antonio, Texas, with patients less than 18 years of age or greater than 80 years of age excluded. Of the 200 patients, 28% were diabetic, 53% hypertensive, and 26% reported sleep apnea. The average age was 50, and patients had a median BMI of 32. Eighty-eight percent (88%) were women. The higher percentage of women studied is attributed to the fact that many of the patients were referred from a rheumatology clinic. Three times as many women suffer from rheumatoid arthritis compared to men.

### 6.4 Statistical analysis

The nose/heart maximum ratios were tested for associations with continuous variables, e.g., BMI, using Pearson correlation coefficient. The direct association of nose/heart maximum ratio with clinical conditions was tested for associations with sleep apnea, hypertension, and diabetes using the Wilcoxon rank-sum test. All testing was two-sided with a significance level of 0.05. Conditions that were individually associated with nose/heart max ratios were entered into a linear regression.

### 6.5 Results

Patients with AD risk factors, including hypertension, diabetes, sleep apnea, BMI > 25, or elevated glucose/HbA1c values had significantly increased nose/heart max ratios on the region of interest analysis of their whole-body blood pool scans (Phillips et al., 2022).

Results are shown in Tables 1–3. The Wilcoxson rank-sum tests of nose/heart max ratios were significantly increased in patients with diabetes (*p* = 0.0020), hypertension (*p* = 0.0123), and sleep apnea (*p* = 0.0002) compared to those without these conditions (Table 1).

**Table 1:** Average values of nose/heart max ratio with different clinical characteristics.

Variable	No	Yes	*p*
Diabetes	0.86	0.96	0.0020*
Hypertension	0.85	0.93	0.0123*
Sleep apnea	0.86	0.99	0.0002*

Values are represented as mean (standard deviation). *p* values depict the Wilcoxon test. * Statistically significant.

**Table 2:** Pearson correlation between continuous variables and nose/heart max ratios.

Variable	Correlation	95% CI	*p*
BMI	0.36	0.23–0.48	<0.0001*
Blood glucose	0.27	0.13–0.39	0.0001*
Hemoglobin A1c	0.25	0.11–0.39	0.0008*
No. of anti-hyperglycemics	0.22	0.08–0.35	0.0021*
No. of anti-hypertensives	0.17	0.03–0.3	0.0192*

*Statistically significant. CI, confidence interval.

**Table 3:** Linear model of nose/heart ratios with diabetes, sleep apnea, and body mass index > 25.

Characteristic	Beta	95% CI	*p*
(Intercept)	0.72	0.65–0.79	<0.001
Diabetes	0.07	0.00–0.15	0.050*
Sleep Apnea	0.09	0.02–0.16	0.015*
BMI >25	0.15	0.07–0.23	<0.001*

*Statistically significant. CI, confidence interval.

Pearson correlation of nose/heart max ratios were significantly correlated with BMI > 25 (*p* < 0.0001), blood glucose levels (*p* = 0.0001), HbA1c (*p* = 0.0008), number of anti-hyperglycemic medications prescribed (*p* = 0.0021) and number of anti-hypertensive medications prescribed (*p* = 0.0192) (Table 2).

Utilizing linear regression analysis, medical conditions associated with AD’s risk factors revealed that a person *without* diabetes, sleep apnea, hypertension, hyperlipidemia, or a BMI > 25 would have an expected nose/heart max ratio of 0.72 or less. Individuals *with* risk factors associated with AD had higher nose/heart max ratios. *The higher the total number* of risk factors for AD, *the higher the nose/heart max ratios*.

The observed increased nose/heart max ratios are as follows: with diabetes (regression coefficient, Beta +0.07, *p* = 0.050), sleep apnea (Beta +0.09, *p* = 0.015), and BMI > 25 (Beta +0.15, *p* < 0.001) (Table 3).

A patient with *two* AD-related conditions, for example, diabetes and a BMI > 25, would have an average nose/heart max ratio of 0.94. The condition of diabetes would statistically add an average of +0.07 and a BMI > 25 would add an average of +0.15. Therefore, a patient with diabetes and an increased BMI > 25 would be expected to have a nose/heart max ratio of 0.72 + 0.07 + 0.15 = 0.94 (Table 3).

**Figure 2:**
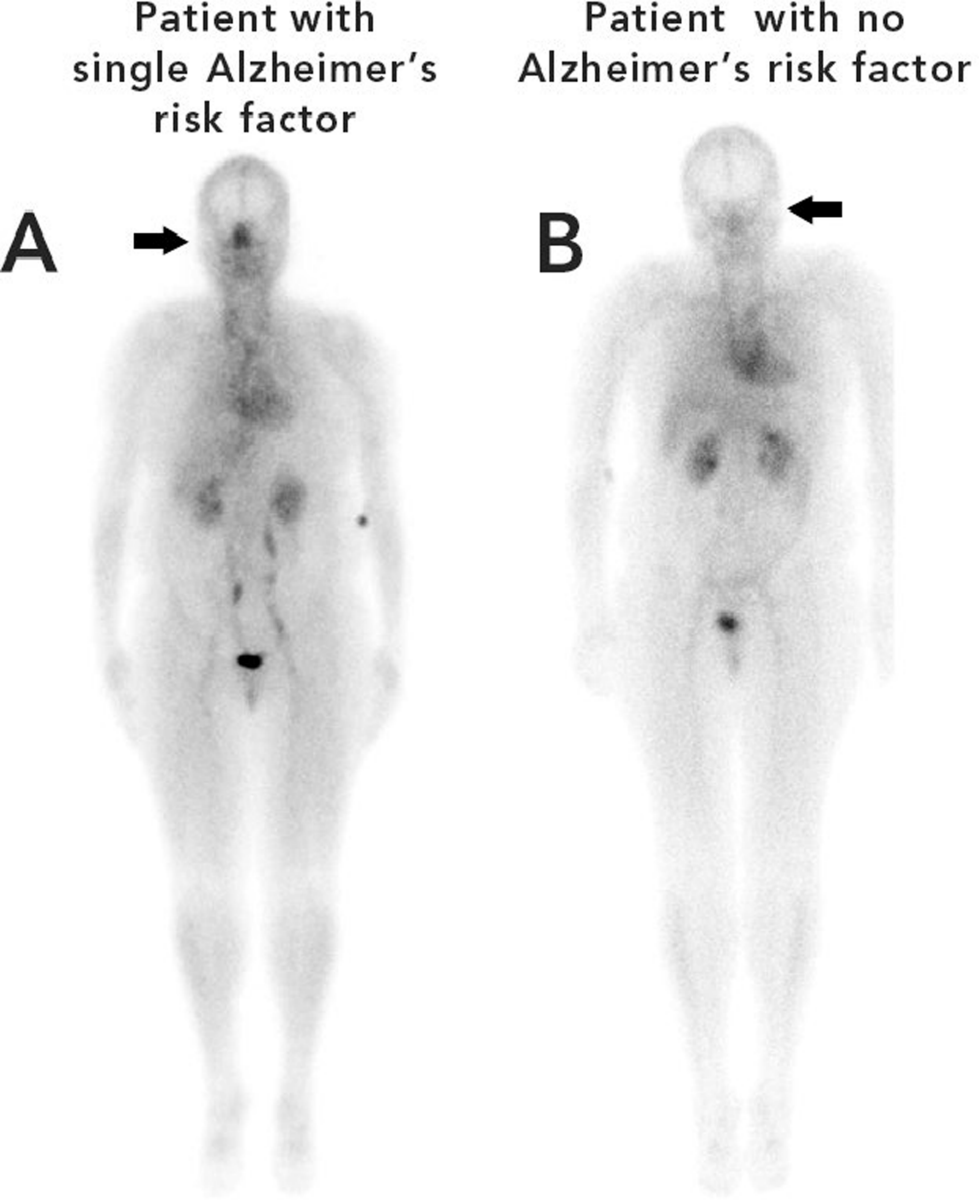
Nuclear images of patient A (left) with a single risk factor for Alzheimer’s disease (sleep apnea) and patient B (right) with no risk factors for Alzheimer’s disease.

Figure 2 is an example of this unique phenomenon. Using scintigraphy whole-body blood pool imaging, a patient with one AD risk factor (sleep apnea) Patient A, is illustrated in contrast to a patient with no AD risk factors, Patient B.

The difference in nasal turbinate vasodilation is easily discernable in Figure 2. The patient with the single risk factor (sleep apnea) had a nose/heart max ratio of 1.16 while the patient with no AD risk factors had a lower nose/heart max ratio of 0.65.

**Figure 3:**
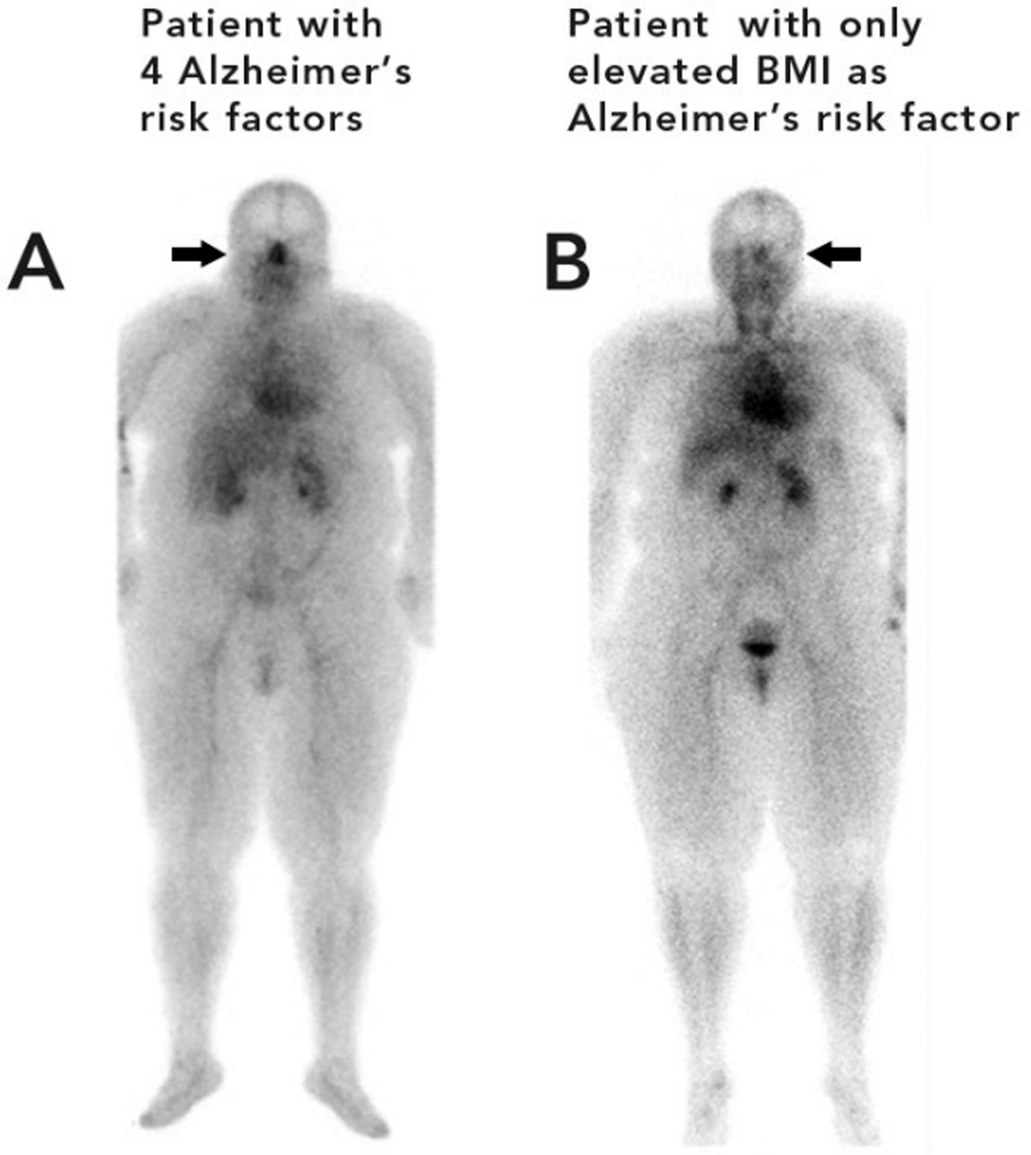
Patient A (left) with four risk factors for Alzheimer’s disease (sleep apnea, diabetes, hypertension, and an elevated BMI) Patient B (right) with one risk factor for Alzheimer’s disease (elevated BMI).

In Figure 3, Patient A had four risk factors for AD, a BMI > 25, sleep apnea, hypertension, and diabetes. Patient A is shown in contrast to Patient B who had only one risk factor, for AD, a BMI > 25.

In Figure 3, the patient on the left (A) with 4 risk factors for AD has noticeably greater nasal turbinate vasodilation when compared with a patient of similar weight with only one risk factor for AD (patient B). The patient with four risk factors for AD had a nose/heart max ratio of 1.22. The patient with only one risk factor for AD had a lower nose/heart max ratio of 0.65.

In both Figures 2, 3, it is easy to visually see the difference in nasal blood pool activity between the two patients. The authors generally found that patients with a higher number of AD risk factors exhibited greater nose/heart max ratios than patients with a similar body habitus and no, or minimal, risk factors for AD. These whole-body blood pool imaging studies have provided insights to the investigators which have led to their proposal of a working hypothesis described in this paper regarding a new causation paradigm for AD and vascular dementias.

## 7 Parasympathetic versus sympathetic nervous systems

The increased nasal turbinate blood pool activity, i.e., nasal turbinate vasodilation, is consistent with upregulation of nasal parasympathetic activity. Increased *parasympathetic* activity results in *nasal turbinate dilation,* while increased *sympathetic activity* or use of sympathomimetic drugs results in *vasoconstriction* of the nasal turbinates (Gainche et al., 2016). This increased nasal turbinate dilation, due to increased parasympathetic activity in patients with risk factors for AD, is surprising considering that essential hypertension, obesity, and sleep apnea are known to be associated with increased sympathetic activity of the cardiovascular system (Narkiewicz and Somers, 1997; Dempsey et al., 2010; Fisher and Paton, 2012; Seravalle and Grassi, 2017, 2022). Increased sympathetic activity in hypertension, a risk factor for AD, is one of the most verified and agreed upon findings in essential hypertension (Smith et al., 2004; Fisher and Paton, 2012; Esler, 2015; Hart, 2016; Grassi et al., 2020).

## 8 Increased parasympathetic activity observed in other organs

In addition to the increased nasal turbinate vasodilation consistent with increased parasympathetic activity that we observed in patients with AD risk factors, we, as well as other researchers, have found evidence of increased parasympathetic activity in other organ systems, including the gastrointestinal system and the parotid salivary glands (Phillips et al., 1991; Schwartz et al., 1995; Lipp et al., 1997; Phillips et al., 1997; Weytjens et al., 1998; Bertin et al., 2001; Watson et al., 2019; Xie et al., 2021; Phillips et al., 2022).

Abnormally *rapid gastric emptying* in hypertensive patients, as previously reported by our group, is consistent with *increased parasympathetic* activity of the upper gastrointestinal system (Phillips et al., 1991; Schwartz et al., 1995; Schwartz et al., 1996; Phillips et al., 1997). The rapid gastric emptying due to increased *parasympathetic* activity has the opposite effect of increased *sympathetic activity* which would *inhibit or slow* gastrointestinal motility.

Our group has also reported increased blood pool activity consistent with vasodilation and increased parasympathetic activity in the parotid glands (Phillips et al., 2022). Physiology studies have shown that vasodilation of the parotid gland is under parasympathetic control (Izumi and Karita, 1994). Increased *sympathetic* activity of the parotid glands causes vasoconstriction of the blood vessels (Garrett, 1975).

In summary, significant evidence now exists to suggest that *parasympathetic activity* can be upregulated in the nasal turbinates, upper gastrointestinal system, and parotid glands in patients with AD risk factors of hypertension, diabetes, and obesity, while these same patients exhibit increased *sympathetic activity* occurring simultaneously in their cardiovascular system (Grassi et al., 2020; Grassi et al., 2023).

## 9 Mechanism of an increase in both parasympathetic and sympathetic activity

What could be the mechanism for this increase in both sympathetic and parasympathetic activity? The authors hypothesize that the increased *parasympathetic activity* in the nasal turbinate, salivary, and gastrointestinal system and increased *sympathetic activity* in the cardiovascular system is due to dysfunction of the glucose regulatory system in patients with Alzheimer’s disease risk factors. Both the parasympathetic and sympathetic systems could be upregulated because the brain glucose sensing system is experiencing a “relative hypoglycemia.”

An elevation of the glucose set point controlled by the brain has been previously proposed by Alonge et al. (2021) in patients with diabetes. This group proposed the brain’s glucose-sensing mechanism becomes dysfunctional in patients with diabetes causing a “relative hypoglycemia” (Schwartz et al., 2023). This “relative hypoglycemia” is thought to be a result of diabetes-associated impairment of the neuronal glucose-sensing process. The authors propose that patients with Alzheimer’s disease risk factors are experiencing a “relative hypoglycemia” with both increased sympathetic and parasympathetic activity. An elevated glucose set-point resulting in a “relative hypoglycemia” would clearly fit with our observation of increased parasympathetic activity in these patients, realizing that an increased rate of gastric emptying is considered to be a significant counter-regulatory response to elevate blood glucose (Schvarcz et al., 1995; Murthy et al., 2021; De Fano et al., 2023). This hypothesis also fits with the well-known increased sympathetic activity of the cardiovascular system in patients with Alzheimer’s risk factors, realizing that increased sympathetic activity is also an important counter-regulatory response to elevate blood glucose levels by increasing liver glucose production via elevated levels of epinephrine, norepinephrine, and glucagon.

Although the mechanism by which the glucose set point becomes elevated leading to autonomic dysregulation is not clearly understood, the authors hypothesize that this autonomic dysregulation is related to the greatly increased intake of ultra-processed foods associated with the modern diet. The modern diet, consisting of ultra-processed products, sucrose, and refined grains, combined with reduced consumption of fiber, fruits, and vegetables, results in significantly elevated postprandial glucose levels leading to an upward resetting of the glucose regulatory system. The continual elevation of blood glucose levels due to the change in diet results in a positive feedback loop allowing more rapid gastric emptying to further increase blood glucose levels. This hypothesis is consistent with the significant increase in obesity which has nearly tripled in prevalence since 1960, and the nearly doubling of the number of patients with hypertension and Alzheimer’s disease over the last 20 years (Mills et al., 2020; Institution, 2024).

The authors’ hypothesis presents a unique mechanism for the etiology of Alzheimer’s disease: resetting of the glucose set-point leads to increasing parasympathetic activity causing increased nasal turbinate vasodilation which obstructs the nasal lymphatic drainage and flow of CSF and its associated waste products through the nasal lymphatics.

## 10 Obstruction of normal nasal turbinate CSF lymphatic clearance

**Figure 4:**
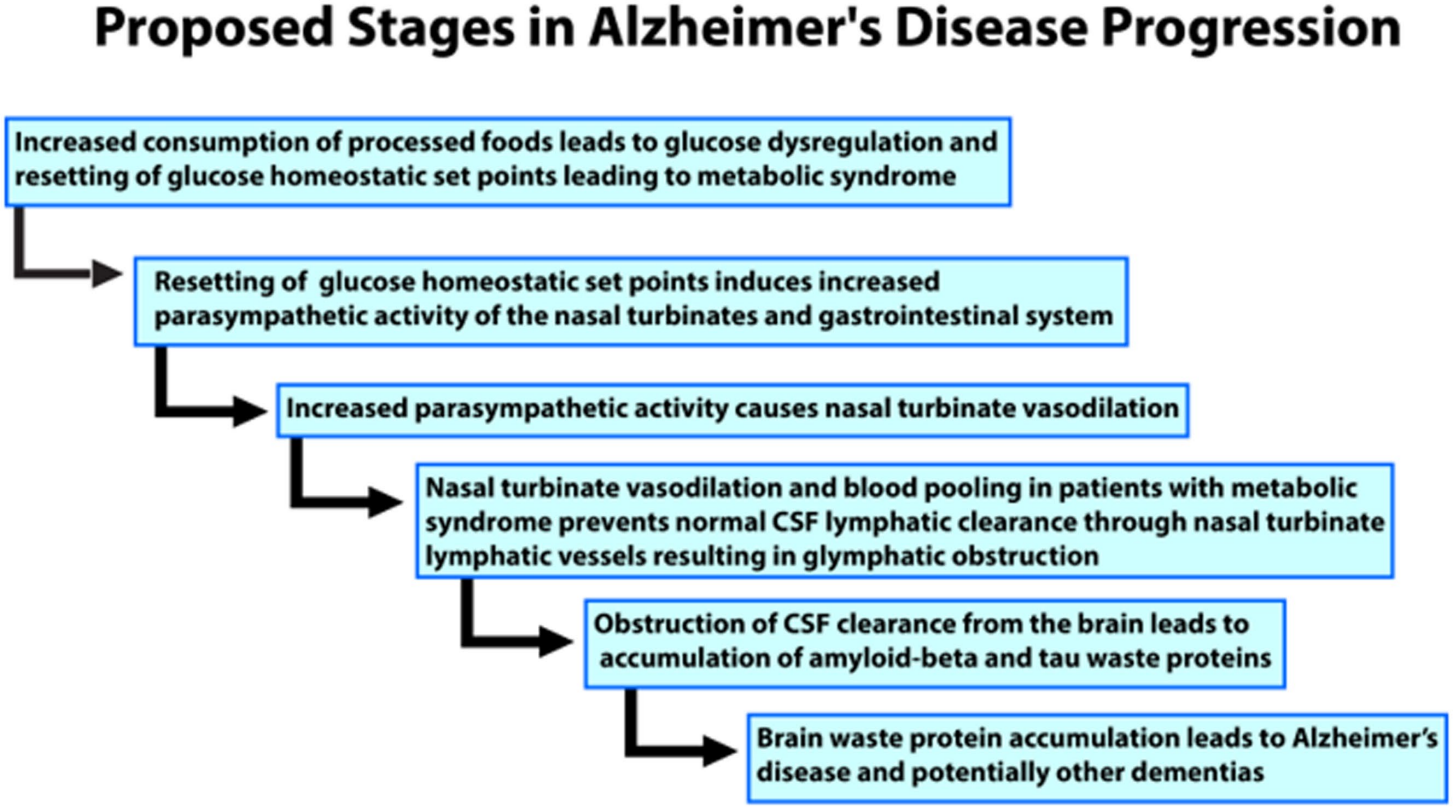
Flow chart of the proposed progress and stages of the authors’ novel hypothesis for the etiology of Alzheimer’s disease.

Prior nuclear medicine research showing nasal turbinate vasodilation in patients with risk factors for AD led to the novel hypothesis for the etiology of AD as diagramed in Figure 4.

We hypothesize that the nasal turbinate vasodilation and resultant blood pooling in patients with AD risk factors leads to obstruction of the normal nasal turbinate CSF lymphatic clearance. This CSF obstruction causes an accumulation of waste proteins, amyloid, and tau, in the brain leading to AD and potentially other dementias such as vascular dementia. The nasal turbinate vasodilation in patients with risk factors for AD is due to increased parasympathetic activity ascribable to dysregulation of the glucose regulatory system under autonomic control.

**Figure 5:**
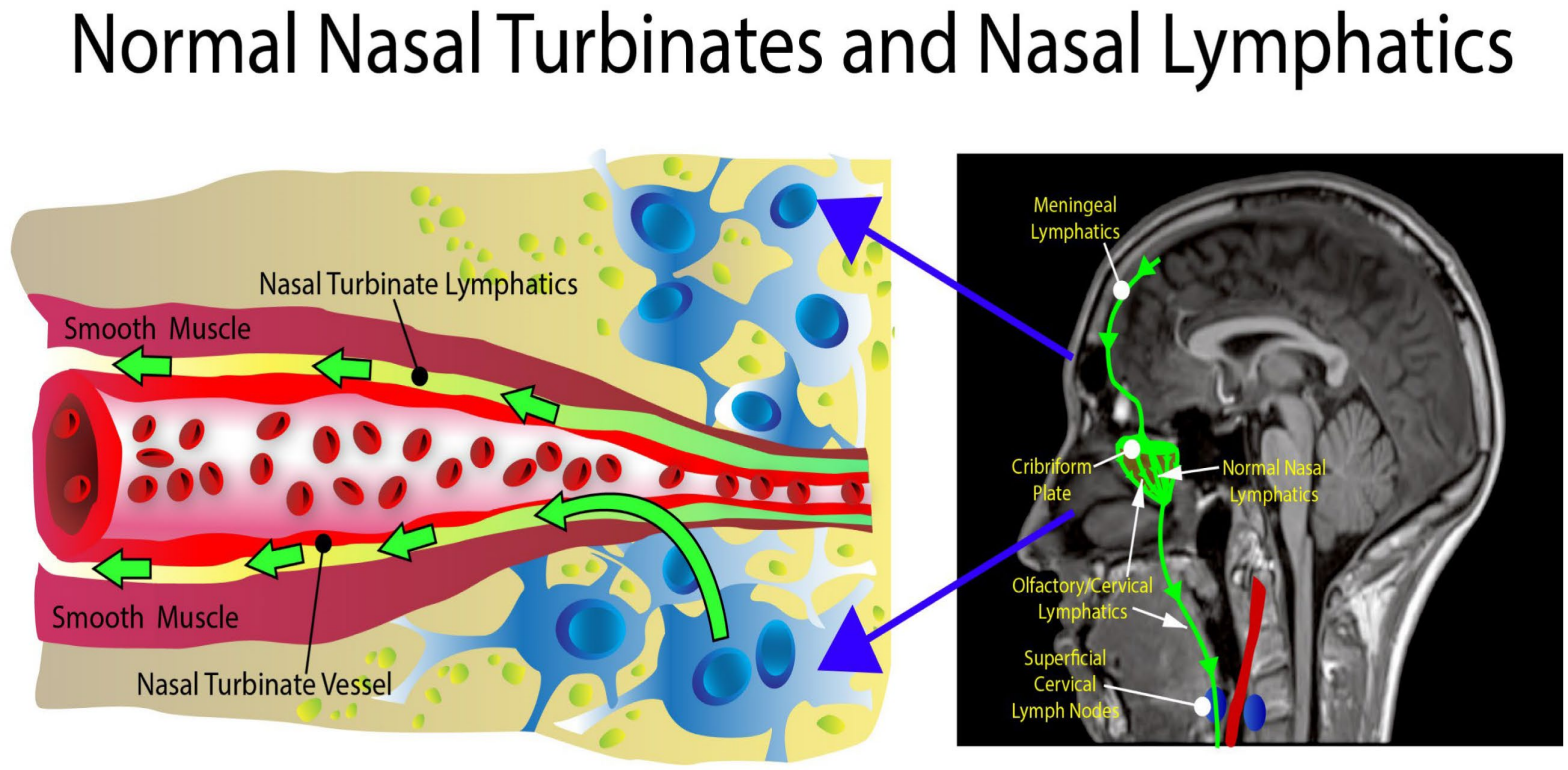
Normal anatomy. There is no obstruction in the area of the nasal turbinates, allowing the nasal turbinate lymphatics to flow freely.

**Figure 6:**
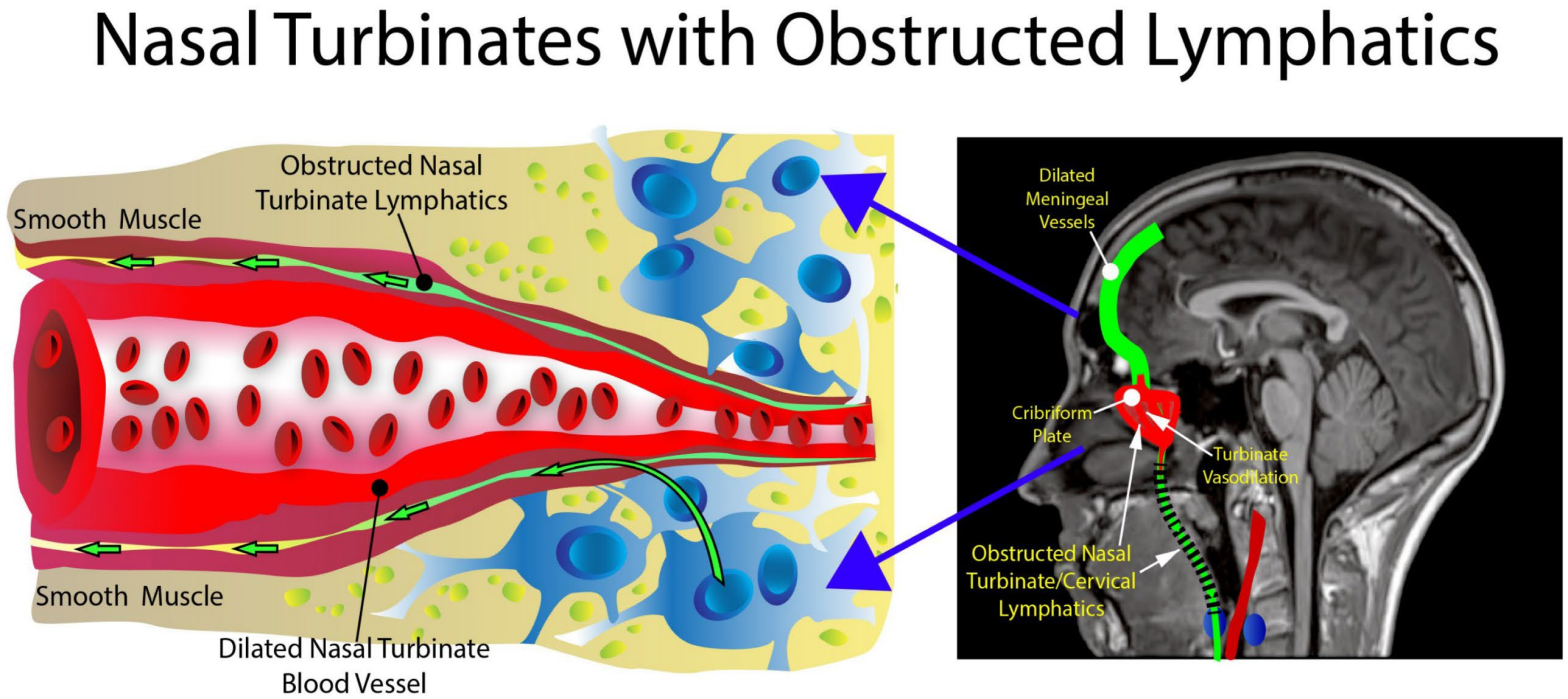
Abnormal anatomy. Vasodilation with blood pooling in the area of the nasal turbinates. The expansion of the vessel results in impingement of the nasal lymphatic flow.

The nasal turbinate vasodilation and blood pooling observed in patients with metabolic syndrome and other AD risk factors obstruct the normal CSF drainage through the nasal turbinates as shown in Figures 5, 6. These figures show how nasal turbinate vasodilation can result in a compression of the surrounding nasal turbinate lymphatics which are responsible for drainage of CSF. Decreased functionality of the lymphatics in multiple regions of the body has been associated with vasodilation due to inflammation (Schwager and Detmar, 2019).

The authors’ hypothesis presents a unique mechanism for the etiology of AD: an increase in parasympathetic activity in the nasal turbinates. The increase in parasympathetic activity causes nasal turbinate vasodilation and blood pooling, obstructing the nasal lymphatic drainage and flow of CSF and its associated waste products through the nasal lymphatics. The increased nasal turbinate vasodilation has been previously described in patients with essential hypertension and other metabolic syndrome features in a recent article by the authors (Phillips et al., 2022).

## 11 Evidence for the hypothesis

Over the past 20 years, significant evidence has been presented to demonstrate that nasal lymphatics are responsible for significant clearance of CSF from the brain. The nasal lymphatics are becoming a more recognized region for the drainage of CSF from the brain. A major proponent of the importance of CSF movement from the subarachnoid space into the nasal turbinate region was Miles Johnston whose work contradicted the most accepted theory—that the majority of CSF is cleared by the arachnoid granulations (Johnston, 2003; Nagra et al., 2006; Koh et al., 2007). As pointed out by Johnston and Papaiconomou (2002), there has been very limited evidence to support the idea that the arachnoid granulations are the primary site of CSF clearance from the brain. There has, however, been significant research supporting the clearance of CSF through the cribriform plate into the nasal turbinate region. In one study, Johnston’s group found that 30 min after injection of radiolabeled human serum albumin into the CSF, the tissue that contained the highest activity was the middle nasal turbinate which had approximately 6 times more activity than the blood (Nagra et al., 2006). In another study, Johnston et al. reported that approximately one-half of a protein tracer was transported from the CSF to the blood via extracranial lymphatic vessels (Boulton et al., 1997). This same group found when CSF transport was blocked through the cribriform plate, resting intracranial pressure doubled from 9.2 cm H_2_O to 18.0 cm H_2_O (Mollanji et al., 2002). A recent review of the importance of nasal lymphatics in CSF clearance has been published and is titled, “The Brain-Nose Interface: A Potential Cerebrospinal Fluid Clearance Site in Humans” (Mehta et al., 2021).

Since an original report by Schwalbe (1869), a large body of work in many different species has indicated a role for lymphatic vessels draining CSF in both cranial and spinal regions. Recently published anatomical and quantitative studies have shown abundant evidence that connections between the CSF and the extracranial lymphatic system represent a significant route for CSF drainage (de Leon et al., 2017; Zhou et al., 2022; Spera et al., 2023; Yoon et al., 2024).

Another recent 2023 study in rats using high-resolution imaging was strongly supportive of lymphatic movement along olfactory nerves. The study concluded that the olfactory nerve pathway into nasal turbinate lymphatics is the major route of CSF clearance from the brain (Spera et al., 2023). In an animal model study by Leeds et al., infusion of Ringer’s lactate with blue dye into the cisterna magna to increase the intracranial pressure caused a 3-fold increase in cervical lymph node flow and an increase in blue-colored nasal discharge that appeared 48 min after the beginning of the infusion (Leeds et al., 1989). The nasal discharge increased from negligible, before the cisternal infusion, to 11.4 mL/h following the infusion. These studies provide support for significant clearance of CSF from the region of the brain into nasal and cervical lymphatics.

Ma et al. found that lymphatic vessels were the major outflow pathway of CSF for both large and small molecular tracers in mice. They also found a significant decline in CSF lymphatic outflow in aged compared to young mice suggesting that the lymphatic system may represent a target for age-associated neurological conditions (Ma et al., 2017). In another recent study by Yoon et al., a nasopharyngeal lymphatic plexus was found to be a hub for CSF drainage to the deep cervical lymph nodes. This plexus was suggested as a possible target for the treatment of age-related neurological conditions which are known to be associated with decreased CSF transport to deep cervical lymph nodes (Yoon et al., 2024).

Meningeal lymphatic vessels located along the dural sinuses have been shown to drain into the cervical lymph nodes (Da Mesquita et al., 2018), and are coupled with, and receive drainage from, the recently described glymphatic system within the brain (Ringstad and Eide, 2024) that was first described by Iliff et al. (2012).

A PET imaging study by de Leon et al. showed tracer activity in the nasal turbinates (de Leon et al., 2017) suggesting CSF movement through the cribriform plate and into the nasal turbinate lymphatics. This study also reported that lateral, ventricle, and superior nasal turbinate CSF clearance abnormalities were found in AD and that ventricular CSF clearance reductions were associated with increased brain amyloid depositions. Consistent with this finding, decreased CSF clearance and increased brain amyloid have been reported in Alzheimer’s disease (Li Y. et al., 2022). Mehta et al. have also recently reviewed the brain-nose interface as a potential CSF clearance site in humans (Mehta et al., 2021).

Disruption of CSF flow through the olfactory system has been proposed as a contributor to the AD pathogenesis (Ethell, 2014). A recent MRI tracer imaging study also provides support for nasal lymphatic obstruction causing impaired peri-olfactory cerebrospinal fluid clearance through the inferior nasal turbinate. This impaired lymphatic clearance through the nasal turbinate was associated with aging, cognitive decline, and decreased sleep quality (Zhou et al., 2022).

## 12 Normal nasal cycle and interaction of nasal turbinate lymphatics

The nasal cycle is the alternating of airflow between nostrils that shifts between the left and right sides over time (Kahana-Zweig et al., 2016). The physical mechanism causing the nasal cycle is due to an asymmetry in blood flow leading to the engorgement of erectile tissue in the inferior turbinate and the anterior part of the nasal septum in one nostril more than the other. This normal asymmetrical enlargement of a nasal turbinate on one side blocks the passage of air. The autonomic nervous system is important in controlling the nasal cycle with sympathetic dominance associated with vasoconstriction and decongestion in one nostril while simultaneous parasympathetic vasodilation and congestion occur in the other nostril (Kahana-Zweig et al., 2016).

The purpose of the nasal cycle has been debated. Some studies suggest the nasal cycle is a method of air conditioning and is useful for removing entrapped contaminants (Soane et al., 2001). Eccles proposed the nasal cycle is a mechanism of respiratory defense against infection with respiratory viruses (Eccles, 2021). It has also been noted that the reciprocal nature of the nasal cycle declines with age (Mirza et al., 1997; Williams and Eccles, 2015) and that the classic nasal cycle may be a marker for age-related central nervous system changes (Mirza et al., 1997). It has been proposed that the periodic congestion and decongestion of nasal venous sinusoids as part of the nasal cycle may provide a pump mechanism for the generation of plasma exudate and that this mechanism is an important component of respiratory defense (Eccles, 1996).

Following thorough research, the authors were unable to find any current studies examining the effect of hypertension, metabolic syndrome, and other risk factors for AD on the nasal cycle. In our nuclear imaging studies, we did not visualize any significant asymmetry, or evidence of a normal nasal cycle, in the distribution of blood between the right and left nasal turbinates in patients with AD risk factors. Conversely, we did notice that patients without risk factors were more likely to have nasal blood pool asymmetry between the right and left nasal turbinates suggestive of a normal nasal cycle. Patients with hypertension and other risk factors for AD in our whole-body blood pool imaging study, who also had a computed tomography (CT) scan of the head, demonstrated symmetrically dilated right and left nasal turbinates without evidence of a normal nasal cycle (unpublished observation).

A malfunction of this normal cycle, with near-permanent vasodilation of the nasal erectile tissue, would result in a blockage of lymphatic outflow from the brain. In this regard, it is interesting that the nasal cycle was found to be diminished with age (Mirza et al., 1997; Williams and Eccles, 2015). In one study, 50% of patients over the age of 70 showed no evidence of a nasal cycle (Williams and Eccles, 2015). Although it has not been previously proposed that the nasal cycle serves as a pump to move lymphatic fluid from the CSF into the head and neck lymphatics, the authors believe that this could be one of the most important functions of the normal nasal cycle. It is, therefore, important to understand the contribution of the nasal cycle to the lymphatic clearance of CSF from the brain via the nasal turbinates.

## 13 Understanding the brain’s glymphatic/lymphatic system

The glymphatic system consists of specialized low-resistance spaces known as Virchow-Robin perivascular spaces that permit CSF inflow deep into the neural parenchyma (Aspelund et al., 2015; Louveau et al., 2015; Nedergaard and Goldman, 2020; Hablitz and Nedergaard, 2021). A detailed review of the glymphatic system has recently been published by the author (W.T.P.) and colleagues (Reiter et al., 2022). The glymphatic system runs in the same direction as blood flow which is propelled by pulsations from the arterial vascular wall. The system can deliver protective molecules, such as melatonin, deep into the brain along the periarterial spaces. It also transports protein waste products, such as amyloid and tau degradation products, from the brain via the perivenous spaces (Nedergaard and Goldman, 2020). The fluid in the perivenous space eventually moves into the subarachnoid space on the surface of the brain where this fluid and any waste material are absorbed into meningeal lymphatic vessels, as reported by Aspelund et al. (2015) and Louveau et al. (2015). This network of meningeal lymphatics serves the same purpose as classical lymphatic drainage and is essential for maintaining neurophysiological homeostasis. The fluid in the meningeal lymphatics is then transported out of the brain and moves into cervical lymphatics. Although the precise anatomic pathway taken by this CSF/lymphatic fluid out of the cranial cavity remains to be clearly defined, the greatest evidence supports its movement along the cranial and spinal nerves, with the olfactory nerve, which is believed to be the most predominant, and its nerve fibers ending in the nasal turbinates (Johnston, 2003; Nagra et al., 2006). Drainage from these meningeal and cervical lymphatics is relatively fast as tracers injected into the brain or CSF accumulate in the cervical lymph nodes within minutes after injection into the brain or CSF (Plog et al., 2015). The discovery of this glymphatic/lymphatic clearance system has clearly shown that CSF and interstitial fluid are directionally transported within the CNS.

### 13.1 Possible obstruction and involvement of the glymphatic/lymphatic system in the pathogenesis of AD

Because the glymphatic/lymphatic system plays a key role in the clearance of amyloid-beta and tau proteins, it has been suggested as a new target to combat neurodegenerative disease (Louveau et al., 2016; Mestre et al., 2020). Failure of the glymphatic system has been described as the final common pathway to the development of dementia (Nedergaard and Goldman, 2020). Many other authors have also suggested a link between the glymphatic system and the pathogenesis of AD (Kopeć et al., 2023; Thipani Madhu et al., 2024).

### 13.2 Impairment of the glymphatic/lymphatic system with age

It has been shown that the glymphatic/lymphatic clearance system is impaired with age. A recent MRI tracer imaging study supporting this theory showed that impaired peri-olfactory cerebrospinal fluid clearance through the inferior turbinate was associated with aging, cognitive decline, and decreased sleep quality (Zhou et al., 2022).

### 13.3 Increased activity of the glymphatic system during sleep

Sleep disturbances are significant risk factors for AD (Borges et al., 2019; Zhang et al., 2022). These sleep disturbances include decreased sleep and sleep apnea. Patients with sleep apnea have been shown to have impaired glymphatic function that may contribute to the increased risk of AD (Roy et al., 2022). Numerous associations have been documented between sleep disturbances and the failure to clear waste products from the brain (Komaroff, 2021). Sleep disturbances are associated with increased CSF metabolite concentrations (e.g., amyloid-beta, orexin, tau proteins) and increased CSF volumes or pressure (Chong et al., 2022). Recent studies have suggested that glymphatic dysfunction is a common underlying etiology of sleep disorders and headache pain (Yi et al., 2022). The glymphatic system is particularly active during sleep whereby potentially toxic neural waste substances that accumulate during wakefulness are cleared via the glymphatic system (Chong et al., 2022; Rasmussen et al., 2022).

It is thought that the brain cell volume decreases during sleep, expanding the size of the perivascular space, and facilitating the influx of CSF into the perivascular space for material exchange and metabolic waste removal (Xie et al., 2013). Animal experiments using intravital 2-photon microscopy in mice showed that glymphatic clearance is decreased by 90% during wakefulness, while protein clearance in the intima of the brain doubles during sleep (Nedergaard and Goldman, 2020; Miyakoshi et al., 2023).

Short sleep duration has also been associated with essential hypertension and other risk factors for AD in many epidemiologic studies (Killick et al., 2023), although there has been no clear pathophysiologic connection found between the two. It is the authors’ hypothesis that decreased CSF clearance due to short sleep and obstructed nasal lymphatics is related to the development of these risk factors.

## 14 Enlarged perivascular spaces found in AD

The perivascular fluid-filled cavities that surround perforating arteries and veins in the brain parenchyma, previously described as Virchow-Robin spaces, play an important role in the glymphatic system. These perivascular spaces can become enlarged and can be detected by special imaging sequences of magnetic resonance imaging (MRI) using diffusion tensor imaging (DTI-ALPS) (Cavallari et al., 2023). In recent years, enlarged perivascular spaces (EPVS) have been linked to an increased risk of cognitive decline, dementia, stroke, and cerebral small vessel disease (Romero et al., 2022; Yang et al., 2023). One study found that EPVS in the hippocampus was associated with the diagnosis of AD (Gertje et al., 2021). EPVS has been proposed as a potential early biomarker of AD (Lynch et al., 2022) even though the cause of these EPVS is unknown. Various speculations concerning the cause of these EPVS have been described including (1) arterial stiffening, (2) protein aggregation, (3) brain atrophy, and (4) destruction of the blood–brain barrier. These proposed mechanisms are still considered to be hypothetical (Yang et al., 2023). Based on our findings, we would propose that obstruction of the nasal turbinate lymphatic drainage system could be a potential mechanism for the enlargement of the perivascular spaces and the development of AD.

## 15 Future confirmatory studies

The proposed mechanism for the development of AD presented in this paper is a working hypothesis and confirmatory studies will be needed. A weakness of the currently presented evidence for this hypothesis is the inability of the authors to follow the patients with nasal turbinate dilatation and increased risk factors for AD over a longer period to observe and document the correlation of nasal turbinate dilation with the possible onset and incidence of AD.

Future prospective studies involving following patients with increased nasal blood pooling over a long period would complement the retrospective studies described in this article. Short-term studies could assess the effect of various interventions to decrease nasal turbinate vasodilation as possible therapies for AD.

Additional areas for future studies include nuclear blood pool imaging for patients with psychiatric and cognitive impaired syndromes to observe if a similar occurrence of nasal vasodilatation and blood pooling would be present. Researchers could follow these patients longitudinally to assess if the NHMR increases over time and correlates with the severity and rate of cognitive impairment. Future studies could also be performed to determine if nasal turbinate vasodilation can provide a predictive marker for the future development of dementia or other neurologic conditions.

An advantage of the nuclear imaging technique is that dynamic imaging can be performed so that changes in the nasal blood pooling are visualized in real time by simply placing a standard gamma camera over the upper body of the patient. The gamma camera can be placed several inches away from the patient resulting in minimal disturbance. This allows studies to be performed during sleep or during other medical or physical interventions that affect the nasal turbinates. To perform these imaging studies for a period of up to 12 h, a blood pool imaging agent such as radiolabeled red blood cells can be utilized to permit dynamic imaging. Technetium-99 m labeled red blood cells are the standard blood pool nuclear imaging agents most commonly used for prolonged imaging of the blood pool. Currently radiolabeled red blood cells are used for locating the site of gastrointestinal bleeding, diagnosing hepatic hemangiomas, and determining left ventricular ejection fractions (Espinosa-Muñoz et al., 2020).

Other imaging studies can also be performed to assess nasal turbinate vasodilation in patients with AD risk factors utilizing MRI or CT, such as those previously reported by Rodrigues et al., who described turbinate hypertrophy in obese patients using CT and analysis of transaxial images (Rodrigues et al., 2022).

Studies can be performed with MRI contrast agents, investigating the lymphatic drainage of cerebrospinal fluid through nasal turbinates and its association with AD risk factors as previously performed by Zhou et al. (2022). Other areas of investigation could include assessing the absence or presence of the nasal cycle in patients with risk factors for AD as compared to controls.

## 16 Potential novel therapeutic approaches for AD that target the nasal turbinate lymphatic drainage

Based on the evidence in this paper, the nasal turbinates are potential targets for the prevention or delay of AD. One possible treatment would be to block the increased parasympathetic activity of the nasal turbinates by blocking the sphenopalatine ganglion that carries parasympathetic activity to the nasal turbinates. The sphenopalatine ganglion is the largest extracranial parasympathetic ganglion of the head (Khan et al., 2014). Sphenopalatine ganglion blockage has been used to treat migraine headaches (Khan et al., 2014) and a recent study has shown that blocking the sphenopalatine ganglion can modestly lower blood pressure (Triantafyllidi et al., 2016). However, completely blocking parasympathetic activity to the nose may not be the best approach for treating those patients with risk factors for AD as it would adversely affect the reciprocal nasal cycle which is dependent on alternating sympathetic and parasympathetic activity to the nasal turbinates (Kahana-Zweig et al., 2016) and, as previously mentioned, could be important for the clearance of CSF fluid from the brain.

Future therapeutic approaches could develop methods to increase the volume of CSF flowing through the nasal lymphatics. The goal of this therapeutic approach would be to restore the normal nasal cycle or to use other medical or physical approaches to increase the movement of CSF through the nasal turbinates and out of the brain region.

Increasing the movement of CSF from the brain region could decrease intracranial pressure and could also lead to significantly more effective treatments for sleep apnea and hypertension.

## 17 Summary and conclusion

This paper describes the observation of significantly increased nasal vasodilation and blood pooling of the nasal turbinates in patients with risk factors for AD which suggests a possible cause for the disease and novel targets for prevention. It is hoped that this article will stimulate future research in this promising area.

## Data Availability

The original contributions presented in the study are included in the article/supplementary material, further inquiries can be directed to the corresponding author/s.
